# The Mediating Effect of Creativity on the Relationship Between Mathematic Achievement and Programming Self-Efficacy

**DOI:** 10.3389/fpsyg.2021.772093

**Published:** 2022-01-10

**Authors:** Jun Liu, Meng Sun, Yue Dong, Fei Xu, Xue Sun, Yan Zhou

**Affiliations:** ^1^College of Education, Capital Normal University, Beijing, China; ^2^Beijing No. 35 High School, Beijing, China; ^3^College of Teacher Education, Capital Normal University, Beijing, China; ^4^Xinyu School Affiliated to Beijing Normal University, Xinyu, China

**Keywords:** programming self-efficacy, mathematic achievement, creativity, mediating effect, upper-secondary school students

## Abstract

**Purpose:** This study aimed to explore the relationship between mathematic achievement and programming self-efficacy, and adopt a mediation model to verify the mediating role of creativity on the relationship between mathematic achievement and programming self-efficacy.

**Methods:** A total of 950 upper-secondary school students were surveyed using their math test scores, the Kirton Adaption-Innovation and the Programmed Self-Efficacy Scale. SPSS-26 was used for descriptive statistical analysis and correlation analysis of related variables. The PROCESS plugin was used to test the mediating effect of creativity.

**Results:** (1) Mathematic achievement has a positive effect on programming self-efficacy, mathematic achievement is positively related to creativity, and creativity also has a positive influence on programming self-efficacy. (2) Creativity has a mediating effect on the relationship between mathematic achievement and programming self-efficacy.

**Conclusion:** The results revealed that mathematic achievement affected programming self-efficacy directly and also indirectly through creativity. This provided certain ideas for the development of programming education for teenagers. Since students’ mathematics learning and creativity are related to programming learning, it is necessary to pay attention to the integration of the disciplines of programming education and mathematics. Further, the cultivation of innovative thinking is also critical to facilitate programming learning.

## Introduction

An increasing number of countries and regions have been attaching great importance to programming education for young people in recent years. In the beginning, most programming students were IT-related professionals who learned programming for their own work purpose. However, the LOGO language was introduced in the field of education which made programming easier for children to learn (Feurzeig et al., 2011). At present, with the development of artificial intelligence (AI), programming has become a significant course for students. Meanwhile, various countries have put forward many specific requirements for programming learning in primary and secondary schools. According to the iDREAMS project, designed by the National Science Foundation, using a Scalable Game Design in the regular school curriculum can improve computer science education (Repenning and Ioannidou, 2008). Moreover, in 2014, the European Commission officially launched Code Week in Europe, with it then receiving widespread attention and continuing to this day (Europe Commission, 2014). In Australia and Turkey, programming education has become an important course in primary and secondary levels (StartupSmart, 2015; Arslan and Tanel, 2020). In recent years, China has also introduced certain educational policies to promote programming education for teenagers in primary and secondary schools. For instance, in 2019, the Ministry of Education of China emphasized that China will popularize programming education at primary and secondary schools (Ministry of Education of the People’s Republic of China, 2019). Meanwhile, several extracurricular programming classes and adaptive programming languages sprang up since the programming learning of teenagers has aroused the concern of society, parents and school (Sun and Zhou, 2019). Hence, it is clear that programming education has attracted significant attention of many countries.

However, several difficulties and challenges emerged as programming education continues to spread among younger students. First, the complexity of programming itself makes some students have a low programming self-efficacy (Davidsson et al., 2010; Hongwarittorrn and Krairit, 2010), which is an important factor affecting the success of students’ programming learning (Altun and Mazman, 2012; Korkmaz, 2012; Tsai, 2019). For most students, learning programming is both complex and challenging, except for those who are interested in and talented at it (Cheng et al., 2013; Akinola, 2015; Kucuk and Sisman, 2017). Altun also pointed out the improvement of programming achievement not only depends on programming cognitive skills and prior knowledge, but also depends on the improvement of programming self-efficacy (Altun and Mazman, 2012). Thus, to improve students’ programming self-efficacy is one of the challenges for programming education. Second, because of abstract thinking and logical reasoning, programming may cause learners great distress which puts forward higher demand for learners’ mathematic achievements (Jenkins, 2002; Garner, 2009; Yukselturk and Altiok, 2017). Many researchers pointed out that math is the foundation of programming learning (Ramalingam and Wiedenbeck, 1998; Rubio et al., 2013). Another study found that students with mathematical thinking, logic and knowledge are more likely to achieve success in programming (Tomai and Reilly, 2014). Therefore, mathematic achievement becomes an important factor affecting programming learning (White, 2003). Third, the goals of programming education for teenagers have changed in intelligent society, because it put forward higher requirements on the innovation ability of talents. Specifically, programming education for teenagers not only emphasizes code writing and program functions, but also creative expression (Dufva, 2018). Creative programming works require students to master programming knowledge and develop innovative thinking in the process of coding (Soykan and Kanbul, 2018; Sandberg, 2019; Fragapane and Standl, 2021). Thus, it can be seen that creativity is an indispensable ability for students’ successful programming.

Although scholars have not directly focused on the complex relationship among mathematics, creativity and programming, some related studies have proved that there may be a certain relationship between them. First, some researchers have explored the relationship between mathematics and programming. They discovered that students’ mathematic achievement could be improved in programming (Friend et al., 2018; Serpe, 2019). However, other studies also found that mathematic learning can promote programming learning, which is an important factor for a programming beginner to learn programming skill (Ramalingam and Wiedenbeck, 1998). Similarly, Tomai and Reilly (2014) revealed that students with math preparation were more likely to successfully complete programming learning. Second, there have been a lot of studies on the relationship between creativity and programming. Most scholars indicated that students can cultivate creativity in programming learning (Kobsiripat, 2015; Noh and Lee, 2020), however, the influence of creativity on programming learning also needs attention. For example, a study by Pardamean et al. (2011) discovered that creativity is essential for learning computer programming. Other studies have also pointed out that programming education not only requires students’ strong programming ability, but also requires them to have strong innovation ability (Zhang and Chen, 2016). Meanwhile, creativity was related to a person’s degree of self-efficacy (Zhang Y. et al., 2018). In summary, there may be a potential influence among mathematic achievement, creativity and programming self-efficacy, but the specific relationship among the three is still unclear, which is worth exploring.

Thus, the purpose of this research is to verify the relationship between mathematic achievement and programming self-efficacy as well as the mediating effect of creativity in this relationship. Understanding the relationship between these three variables is conducive to clarifying the direction of adolescent programming education reform from a more comprehensive perspective, breaking through the current difficulties and challenges, and realizing the development of adolescent programming education.

## Literature Review and Hypotheses

### Programming Self-Efficacy and Mathematic Achievement

*Self-efficacy* refers to an individual’s expectations and judgments of their own specific behaviors or abilities to achieve a desired goal (Bandura, 1977). *Programming self-efficacy* refers to the confidence of students in their ability to learn and complete programming tasks (Compeau and Higgins, 1995; Rohatgi et al., 2016). There are two descriptions of self-efficacy in programming education. First, there is computer self-efficacy, which refers to a person’s judgments of their own ability to use a computer (Compeau and Higgins, 1995). Second, there is computer programming self-efficacy (CPSE). The Computer Programming Self-Efficacy Scale (CPSES) used to measure CPSE, as developed by Ramalingam and Wiedenbeck (1998), is one of the most frequently used tools for measuring programming self-efficacy. Related to this research, studies have focused on the measurement of students’ programming self-efficacy and its influencing factors. One study found that using Visual Programming Language to intervene in students’ programming lessons can reduce the difficulty of programming learning, while also improving their self-efficacy in this area (Tsai, 2019). Simultaneously, there is also a relationship between computer programming self-efficacy and programming learning (Abdunabi et al., 2019).

*Academic achievement* refers to the level of knowledge and skills acquired by students after learning the contents of a certain subject (Zimmerman, 1990; Zhou et al., 2006). According to this concept, it can be concluded that *mathematic achievement* refers to the level and skills of students after a period of mathematical learning. Students’ mathematic achievement can be generally represented by their math scores in some key math exams, such as mid-term and final exams (Jacob, 2012; Yaratan and Kasapoğlu, 2012; Buzzai et al., 2020). According to the research, the change of students’ math scores represents their growth in mathematic achievement (Scammacca et al., 2020).

There may be a connection between programming and mathematical learning. On the one hand, it has been proved by many studies that self-efficacy can affect academic performance (Ayotola and Adedeji, 2009; Affuso et al., 2017), but some studies also revealed that academic performance can predict self-efficacy (Heggestad and Kanfer, 2005; Mijung et al., 2018). As Hwang et al. (2016) found, there is an interactive relationship between self-efficacy and academic performance, that is, the impact of past academic performance on self-efficacy is greater than that of self-efficacy on academic performance. This suggests that it is feasible to predict programming self-efficacy with mathematic achievement. On the other hand, programming requires both logical reasoning and problem-solving abilities (Govender, 2007). These two are also important skills in the field of mathematics, which are directly reflected in the mathematic achievement of learners (Bocconi et al., 2018). A study by Mathews (2017) suggested that mathematical achievement would affect the success of programming learning. Another research confirmed that students’ mathematic achievement is positively correlated with their levels in programming courses (White and Sivitanides, 2003). However, the predictive relationship between mathematic achievement and programming self-efficacy is unclear. Therefore, we propose the following hypothesis:

H1: Mathematic achievement has a positive effect on students’ programming self-efficacy.

### Creativity and Programming Self-Efficacy

Research on *creativity* can be traced back to Kirton’s discussion about adapters and innovators (Kirton, 1976, 1978). In this theory, Kirton divides people into innovators and adaptors, according to the cognitive style adopted by them. Adaptors like to follow the rules, whereas innovators cannot stand routine and tend to be creative. At present, several researchers believe that creativity is a highly important skill (Pink, 2006; Van Harpen and Sriraman, 2013; Henriksen et al., 2019; Bicer et al., 2020). Simultaneously, studies have shown that a positive creative atmosphere contributes to students’ cultivation of overall creative abilities (Liu et al., 2018).

The current international programming education for teenagers calls for attention to the influence of creativity on programming learning (Fragapane and Standl, 2021). Existing researches claimed there is a strong link between creativity and programming learning. Perez-Poch et al. (2016) discovered that high levels of creativity are associated with the excellence of learners in programming. Giannakos et al. (2013) proposed that developing creativity is an excellent way to promote and teach programming. Furtherly, Pardamean et al. (2011) revealed that it is very valuable for future research to explore the forecasting effect of creative thinking on students’ programming education. Although many researchers have studied the relationship between creativity and programming education, the specific effect between creativity and programming self-efficacy is still not clear. Hence, we proposed a second hypothesis:

H2: Creativity has a positive effect on students’ programming self-efficacy.

### Mathematic Achievement and Creativity

Creativity is regarded as an important factor closely related to mathematic achievement, and many scholars attached importance to it. Previous works have demonstrated that mathematic education has an effect on students’ creativity (Hwang et al., 2005; Livne and Milgram, 2006; Jeon et al., 2011; Kattou et al., 2013). Shen (2014) also indicated that students’ creativity can be interpreted and enhanced in math learning. A survey of junior middle-school students also indicated that there is a general difference in scientific creation among students with poor, average and excellent mathematics scores (Jin, 2006). This means that students’ mathematic achievement also has an impact on their creativity. Thus, this led us to propose a third hypothesis:

H3: Mathematic achievement has a positive effect on students’ creativity.

### Mediating Effect

The aforementioned studies showed that students’ mathematic achievement is related to creativity, and creativity is also closely related to programming learning, which indicate that there may be a close relationship among them. The related research provides important enlightenment for us to study the relationship among the three. For instance, there have been studies exploring the relationship between creativity, computer science and mathematics (Erdogan et al., 2008). Similarly, researchers have examined the relationship between the use of computer technology in interdisciplinary learning (STEM subjects) and creativity (Kuo et al., 2019). More importantly, as Kastl et al. (2017) proposed, students need mathematical knowledge to create programming works, as well as creative exposition. Pasini et al. (2017) also suggested that math learners’ creative thinking has an impact on their programming performance. These studies provide clues for us to explore how mathematic achievement and creativity affect programming self-efficacy. Creativity may be a bridging factor between math learning and programming education. Hence, this study hypothesized the following:

H4: Creativity plays a mediating role between mathematic achievement’ s relationship with students’ programming self-efficacy.

**FIGURE 1:**
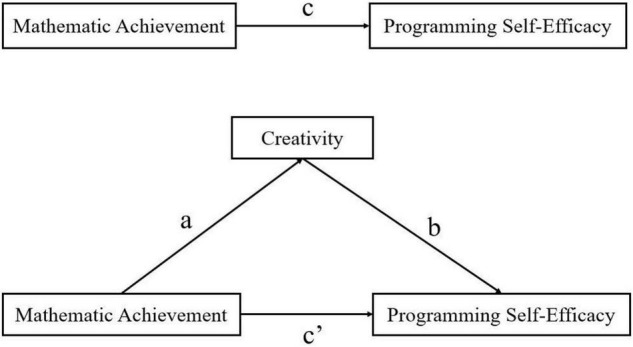
The mediation model of creativity.

In view of this hypothesis, this study will build a model and verify the mediating effect (see Figure 1).

## Materials and Methods

### Participants

The research participants were selected from a group of students with programming experience in high schools in Beijing, China. A total of 1,019 students from four different high schools took part in the survey. The participants who are ranging in age from 16 to 18, had one or two 45-min programming lessons a week at school. In addition, while taking into account the differences between the four schools, this study adopted a proportional random sampling method, with a 3:1 ratio of urban and suburban areas, and then selected four regions in Beijing, China.

On the day of data collection, students and their guardians provided written informed consent. students were told that their participation was voluntary and that there would be no consequences if they decided not to participate in the study. None of the participants were blind to the nature and contents of the experiment. Additionally, there were professional teachers present, who helped students when they faced problems while filling in the questionnaire. This ensured that participants were explicitly involved in the study.

After removing any incomplete responses, the data came to 950 (93.23%) usable surveys. The statistical collation results are shown in Table 1. Of the 950 respondents, 504 (53.1%) were female and 446 (46.9%) were male. According to the school areas, 201 students were studying in the Xicheng (urban) District (21.16%), 105 were studying in the Haidian (urban) District (11.05%), 210 were studying in the Chaoyang (urban) District (22.11%), and 434 were studying in the Shunyi (suburban) District (45.68%).

**TABLE 1:** Descriptive statistical analysis for the observed variables.

Variables	Number of samples	Mean	*SD*
MA	950	3.100	1.760
PSE	950	3.237	1.128
Creativity	950	3.480	0.766

*MA, mathematic achievement. PSE, programming self-efficacy.*

### Materials

The questionnaire used to collect data in this study contained three sections. In the first section, we collected demographic information, including participants’ gender, age and certain family information. Moreover, with the consent of both teachers and students, we obtained students’ recent mid-term math scores, including the specific scores and the levels. Considering that the experiment was carried out in different schools, math grades were based on their total scores and were divided into five levels. Scores ranging from 120–150, 105–119, 90–104, to 75–89 were classified as levels 5, 4, 3 and 2 respectively; scores under 75 indicated Level 1. In this way, students’ mathematic achievements were outlined. In order to ensure the accuracy of the participant’s recall of their math scores and grades, we actively communicated with teachers to ensure that the questionnaires were administered 1 week after the midterm exam. In addition, in order to ensure the reliability of the research data, the existing data were strictly screened and invalid and/or problematic data were removed.

The second section included the Programming Self-Efficacy Scale. Finally, the last section collected the Kirton Adaption-Innovation (KAI). The two scales used in this study were derived from their English versions and were translated using the back-translation method (Brislin, 1970). The back-translation method improves the accuracy of a given translation and involves the following process: a researcher translates the scale from English to Chinese, then a second researcher translates it back from the Chinese version and creates a reverse translated version. In the end, a third researcher compares and checks the three versions (the original, translated, and reverse translated versions) to ensure that there is equivalence between the original English and the translated Chinese versions.

The Programming Self-Efficacy Scale was initially developed by Kukul et al. (2017) to measure the programming self-efficacy of secondary school students. Afterward, this scale was adapted by Soykan and Kanbul (2018) to survey K12 students’ coding self-efficacy. Therefore, this scale was used in this study to collect the computer programming self-efficacy of high school students. This scale consists of 31 items, measured on 5-point Likert-type scales, ranging from 1 point for “strongly disagree” to 5 points for “strongly agree”. A score ranging from 1 to 2.49 means that one has low self-efficacy, with an average self-efficacy being measured at 2.50–3.49 points. Finally, achieving 3.50 points and higher reflects a high degree of self-efficacy. The Cronbach’s alpha of the Programming Self-Efficacy Scale is 0.988, meaning that it has a good reliability. The sample items include: “When encountering a problem, I can distinguish whether the problem is suitable for programming,” “I can break complex programming problems into small problems to solve,” “I can choose the best solution to a programming problem.”

The KAI developed by Kirton (1976) was used to measure students’ creativity. Although KAI is primarily used to measure the style of creativity, which classifies learners as adaptors and innovators, several studies have reported a strong correlation between style of creativity and creativity level (Puccio and Chimento, 2001; Phelan and Young, 2003; Ee et al., 2007; Puccio et al., 2011). For example, the innovators generally have higher innovative thinking and creativity (Camacho-Minano and del Campo, 2017; Zhang H. et al., 2018). Therefore, KAI was used in this study to test students’ creativity. The KAI consists of 32 items measured on a 5-point Likert-type scale, ranging from “strongly disagree” to “strongly agree.” Finally, the average score is calculated and the higher the score, the higher the creativity. Cronbach’ s alpha of the KAI was 0.976, which indicated good reliability. The sample items include: “I have a lot of ideas when I encounter problems or things,” “I think it is easier to create something new than to improve on something that is already there.”

### Data Analysis

SPSS-26 and the PROCESS plugin (version 3.3) were used to analyze the data. First, following a reliability and validity analysis, a descriptive analysis was carried out using SPSS. Second, a Pearson product-moment correlation analysis was used to check the relationships between mathematic achievement, programming self-efficacy and creativity, and a coefficient of variance inflation test was conducted. Finally, a mediation analysis, using the PROCESS plugin in SPSS, was conducted to explore the mediating effect of creativity, and to test our four hypotheses. The causal steps published by Baron and Kenny (1986) and the steps of the causal effect test summarized by Wen et al. (2004) were mainly used for testing the mediating effect. The method proposed by Baron and Kenny is popular for testing the mediating effect. However, some doubts about the causal steps arose after an increasing number of experts studied the method of the mediating effect test. Wen et al. (2004) verified several popular and effective methods and finally came up with a clear mediation effect testing method. His method was adopted in this study.

## Results

### Descriptive Statistical Analysis and Correlation Analysis

Table 1 contains the descriptive analysis results of the students’ mathematic achievement, programming self-efficacy, and creativity. As demonstrated in the table, the mean of mathematic achievement was 3.100 and the standard deviation was 1.760; the mean of programming self-efficacy was 3.237 and the standard deviation was 1.128. The mean of creativity was 3.480 and the standard deviation was 0.766.

Next, the correlation analysis among these three variables was conducted by calculating the Pearson product-moment correlation coefficient (see Table 2). First, mathematic achievement had a positive impact on programming self-efficacy, with a significant correlation (*r* = 0.073, *p* < 0.05). Second, a strong positive correlation was found between creativity and programming self-efficacy [*r* = 0.746, *p* < 0.001, VIF = 1.000 < 10 (there was no multicollinearity)]. Third, there was another significant positive correlation between mathematic achievement and creativity (*r* = 0.084, *p* < 0.01).

**TABLE 2:** Pearson correlations for the observed variables.

Variables	MA	PSE	Creativity
MA	1		
PSE	0.073**	1	
Creativity	0.084**	0.746**	1

MA, mathematic achievement; PSE, programming self-efficacy. *p < 0.05; **p < 0.01.

### Mediation Analysis

The final hypothesis of this study involved examining the mediating effect of creativity. The PROCESS plugin (version 3.3) was used to analyze its mediating effect with mathematic achievement as the independent variable, programming self-efficacy as the dependent variable, and creativity as the mediating variable. The model (Model 4) was used to identify the role of creativity in the relationship between mathematic achievement and programming self-efficacy. The results (see Table 3) show that mathematic achievement significantly predicts programming self-efficacy (*B* = 0.073, *t* = 2.259, *p* < 0.05). In addition, mathematic achievement has a significant positive predictive effect on creativity (*B* = 0.084, *t* = 2.599, *p* < 0.01). Furthermore, creativity has a significant positive predictive effect on programming self-efficacy (*B* = 0.746, *t* = 34.350, *p* < 0.001). This means that, in the model (see Figure 1), there is a significant positive correlation in paths a–c. However, the direct influence of mathematic achievement on programming self-efficacy is not significant (*B* = 0.011, *t* = 0.482). We used the bias-corrected percentile Bootstrap method as proposed by Wen et al. (2004) to test the mediating effect. The Bootstrap test results indicated that the mediating effect of creativity had bootstrap confidence intervals (95%) with no zero between their lower and upper limits, while the direct effect of mathematic achievement on programming self-efficacy had bootstrap confidence intervals (95%) with zero between their lower and upper limits. This suggests that the model includes significant indirect effects, with the direct effect accounting for 14.33% and the indirect effect accounting for 85.67% (see Table 4).

**TABLE 3:** Mediation analysis results for the observed variables.

Regression equation		Fitting indices				Significance
Outcome variables	Predictor variables	*R*	*R* ^2^	*F*(*df*)	Â	*T*
Creativity	MA	0.084	0.007	6.755**	0.084	2.599**
PSE	Creativity	0.746	0.557	595.674***	0.746	34.350***
	MA				0.011	0.482
PSE	MA	0.073	0.005	5.103*	0.073	2.259*

MA, mathematic achievement; PSE, programming self-efficacy. *p < 0.05; **p < 0.01; ***p < 0.001.

**TABLE 4:** Total, direct and indirect effects among the variables.

	Effect size	Boot SE	Boot CI lower limit	Boot CI upper limit	Relative effect size
Total effect	0.0586	0.0261	0.0078	0.1107	100.00%
Direct effect	0.0084	0.0181	−0.0268	0.0437	14.33%
Indirect effect	0.0502	0.0192	0.0125	0.088	85.67%

*Boot SE, boot standard error; Boot CI, boot confidence interval; Lower limit and upper limit, the lower limit and upper limit of the 95% boot confidence interval; Relative Effect Size, direct effect size or indirect effect size divided by the total effect size.*

## Discussion

### Discussion of the Results

The results of this study support its hypotheses and are consistent with the findings of previous research.

Firstly, the study results support its first hypothesis in that they reveal a significantly positive correlation between mathematic achievement and programming self-efficacy. As shown in the “Results” section, there is a high or low correlation among these variables, as well as a relatively small effect size. This is due to the peculiar context. Researchers have indicated that computer science is largely a field of applied mathematical principles (Papert, 1972), and that there is a strong link between maths and programming (Sauter, 1986). Research has revealed that there is a connection between mathematics and programming, which has a lot to do with the nature of the subject itself. Mathematical ability has been considered as one of the necessary abilities for programming learning activities. The abilities of reasoning, logical thinking and basic computing required in programming learning all need a mathematical base. Mathematics not only represents a kind of ability, but also a way of thinking (Liu, 2019). This is in alignment with the study conducted by Kattou et al. (2013), which stated that “computer science is evolving along mathematical lines;” this statement shows how important mathematic abilities are for computer science students (Fajrina et al., 2020). Certain computer science educators have also explained this by outlining that mathematical thinking is a method of computer education (Mcmaster et al., 2010). This means that mathematics is a necessary ability for programming learning, because the inherent reasoning, logical thinking, and problem-solving abilities required by programming all rely on mathematics. Additionally, the positive correlation between mathematic and programming scores has also been verified (Razak and Ismail, 2018). This suggests that students who perform well in maths are more likely to have a higher programming self-efficacy. Therefore, many researchers have developed programming courses that integrate mathematical thinking to ensure that students achieve a higher level of programming self-efficacy through the process of using their mathematical thinking skills (Soloway, 1993; Taylor et al., 2010).

Secondly, the results of this study support its second hypothesis that states that there is a significantly positive correlation between creativity and programming self-efficacy. First, (Kirton, 1976) analyzed the differences between innovator and adaptor thinking styles. Innovators tend to believe that novel talents are possessed by people with the “correct” form of creativity, and thus they tend to have a higher programming self-efficacy. This can be explained by the fact that innovators tend to maintain a higher programming self-efficacy when learning this skill. Creativity is crucial to computer learning (Liu et al., 2011) because students cannot learn computer programming without forming creative new ideas (Kobsiripat, 2015). Furthermore, one study revealed that high levels of creativity are associated with superior programming achievements (Perez-Poch et al., 2016).

Thirdly, the results of this study support its third hypothesis that states that there is a significant correlation between mathematic academic achievement and creativity. We can analyze the reasons for this conclusion from examining the characteristics of creativity. First, both innovators and adaptors are achievement-oriented in their learning, but they have different goals. Innovators are more pioneering and divergent in their goal setting, aiming at proficiency, while adaptors are more risk-averse and have clearer self-goals (Ee et al., 2007). Simultaneously, as mentioned earlier, mathematical learning requires more logical thinking and divergent abilities, which is consistent with the goals of innovators. Leikin (2007) also indicated, in their study, that students were more likely to generate innovative ideas from multiple perspectives when solving complex mathematical problems. Several math competitions are aimed at cultivating students’ creativity in the process of applying mathematical knowledge (Kovari and Rajcsanyi-Molnar, 2020). It is generally believed that students with higher mathematic academic performance are more creative than those with lower performances in this area (Manchanda and Sood, 2012). Meanwhile, other researchers have demonstrated a significant positive correlation between math achievement and creativity. Therefore, students with higher mathematical achievements are more likely to be trailblazers and innovators.

Fourthly, the findings of this study also support its fourth hypothesis, which states that creativity plays a mediating role in the relationship between mathematic achievement and programming self-efficacy, which is consistent with the findings of prior research. First, students with higher mathematic achievements tend to be more creative, which is related to the flexibility and novelty inherent in mathematical thinking (Ayllón et al., 2016). Second, creativity is essential in learning computer programming. As such, there is a connection between creativity and the two variables analyzed in this study. Finally, we verified that the direct effect of mathematic achievement and programming self-efficacy was not significant, but that the effect is caused by creativity as a mediating variable. For example, students will constantly develop their creativity in the process of mathematical learning and will need to solve complex logical problems from multiple perspectives (Leikin, 2007). Simultaneously, programming is also a process of design creation and imagination (Romero et al., 2017). Learning computer programming can thus help students to understand mathematical concepts, as well as develop both logical thinking and creativity, thus enabling them to have a higher degree of programming self-efficacy (Park and Hong, 2009). Therefore, mathematic achievement, creativity, and programming self-efficacy are closely related.

### Implications

In a theoretical sense, this study complements previous research which examined the relationship between mathematics, programming learning, and creativity. On one hand, this study comprehensively explored the positive influence between mathematic achievement and programming self-efficacy. Additionally, the study also tested the mediating effect of creativity through a mediation analysis. This provides theoretical support and inspiration for an integrated study design of mathematics and programming education in the future.

In a more practical sense, this study provides a new way of thinking for the interdisciplinary curriculum integration and teaching design of programming education. First, interesting programming languages should be fully utilized to stimulate K-12 students’ interest, and the integration of mathematics and programming education should be strengthened. This can help cultivate students’ logical analysis and problem-solving abilities, which are also needed in mathematic learning. Second, students’ personalities and characteristics should be considered in programming education. Teachers should get to know students and improve their teaching skills from the perspective of those learning. Teachers provide different teaching resources and teaching strategies for students with different levels of creativity, so as to promote a better teaching effect. In programming education, teachers can help students’ programming self-efficacy through innovative learning activities to cultivate students’ confidence and interest in programming. The most important thing is that teachers should constantly explore the links between disciplines and programming education, strengthen the integration of disciplines and cultivate students’ higher-order thinking in teaching practice.

### Limitations and Directions for Future Research

This study has some limitations. First, the research participants of this study were all upper-secondary high-school students in Beijing, which is one of the regions with the fastest development of and superior conditions enabling programming education. This may influence the generalization performance of the findings. In future studies, students from other regions and learning stages should be involved. Second, this study utilized a cross-sectional design, meaning that long-term research is required in future. Third, the measurement of students’ creativity in this study can adopt more diversified measurement tools. For example, use KAI for style differentiation of creativity, and combine with the Torrance Tests of Creative Thinking (TTCT; Torrance, 1974) to comprehensively measure students’ creativity performance.

As for the future research, first of all, how to better improve the effect of programming education through the role of mathematics and creativity needs further research. Because this study results indicate that mathematic achievement and creativity can affect programming self-efficacy, and programming self-efficacy is crucial to programming education. Thus, how teachers and students actually use this relationship to improve students’ programming self-efficacy and promote programming learning needs to be considered. Second, there may still be a number of factors that have not been considered in this study. In future studies, other possible variables, such as learning methods and interest in programming, should be studied in the area of adolescent programming. More importantly, with the new requirements in the development of education in the current era, programming and innovative education methods are gaining greater levels of importance in the field of teaching. In future, the methods to promote programming education and innovative development more effectively is a topic worth exploring.

## Conclusion

In the present era of AI, programming has become a crucial skill closely related to people’ s life and survival. In recent years, a number of policies have promoted the popularization of programming education from a younger age. However, programming learning itself has certain difficulties, especially for young programming students. Therefore, it is extremely important to know how to effectively improve students’ programming learning. As one of the important factors in programming learning, programming self-efficacy needs attention. Considering the possible effects of both mathematics and creativity on programming, this study established a model to explore the relationship among mathematic achievement, programming self-efficacy and creativity. To conclude, the correlation analysis suggests that mathematic achievement has a positive effect on programming self-efficacy. Further, the mediation analysis confirmed the mediating role of creativity in this relationship. Due to the mediation of creativity, students with higher achievements in mathematics tend to have a higher programming self-efficacy. The results of this study provide insights for the future development of programming education for adolescents. Both teachers and students need to pay attention to the integration of subjects and the cultivation of higher order thinking, with the help of mathematical and innovative thinking to promote programming learning.

## Data Availability Statement

The original contributions presented in the study are included in the article/supplementary material, further inquiries can be directed to the corresponding author.

## Ethics Statement

The studies involving human participants were reviewed and approved by the Ethics Committee of Capital Normal University. Written informed consent to participate in this study was provided by the participants’ legal guardian/next of kin.

## Author Contributions

JL and YZ designed the research. MS, YD, and FX performed the literature search and data analysis. JL, YZ, and XS reviewed and revised the manuscript. All the authors have commented on previous editions, read, and approved the final draft of the manuscript.

## Conflict of Interest

The authors declare that the research was conducted in the absence of any commercial or financial relationships that could be construed as a potential conflict of interest.

## Publisher’s Note

All claims expressed in this article are solely those of the authors and do not necessarily represent those of their affiliated organizations, or those of the publisher, the editors and the reviewers. Any product that may be evaluated in this article, or claim that may be made by its manufacturer, is not guaranteed or endorsed by the publisher.
